# Reliability and validity of the Chinese post-discharge coping difficulty scale-parent form in parents of premature infants: a multicenter cross-sectional study

**DOI:** 10.3389/fpsyg.2023.1095485

**Published:** 2023-09-06

**Authors:** Jin Liu, Jingjing Gong, Qingqing Shen, Zaixia Si, Jianli Gao, Leijie Xu, Xiaoqin Liu, Yayun Song, Minmin Li

**Affiliations:** ^1^Department of Neonatology, Shandong Provincial Hospital Affiliated to Shandong First Medical University, Jinan, China; ^2^Department of Neonatology, Linyi People's Hospital, Linyi, China; ^3^Department of Neonatology, Maternity and Child Care Center of Dezhou, Dezhou, China; ^4^Department of Neonatology, Weifang Maternal and Child Health Hospital, Weifang, China

**Keywords:** Chinese, post-discharge coping difficulty, parent, premature infants, neonatal nursing, validity, reliability

## Abstract

**Background:**

The measurement of the coping difficulties of parents of premature infants after discharge provides objective data for nurses to prepare infants for discharge. However, no Chinese scale has been developed to measure parents’ coping difficulties after their premature infants are discharged.

**Aim:**

To translate the parent version of the Post-Discharge Coping Difficulty Scale (Ped-PDCDS) from English to Chinese and test the reliability and validity of the Chinese version in parents of premature infants.

**Methods:**

A multicenter cross-sectional study of 356 parents of premature infants was conducted. The scale was symmetrically translated. Validity was evaluated in terms of content, construct, discriminant, and convergent validities. Reliability was assessed in terms of internal consistency, split-half reliability, and test–retest reliability.

**Results:**

The Chinese Ped-PDCDS finally contained 11 items. Exploratory and confirmatory factor analyses results showed that the Chinese Ped-PDCDS had three dimensions, and the convergent and discriminant validities of the scale was satisfactory. The overall reliability, split-half reliability, and test–retest reliability of the scale was 0.85, 0.92, and 0.84, respectively.

**Conclusion:**

The Chinese Ped-PDCDS has adequate psychometric properties, and is an easy and appropriate instrument for measuring parents’ difficulty in coping with premature infants.

## 1. Introduction

According to statistics, approximately 15 million preterm infants are born worldwide every year (March of Dimes, PMNCH, Save The Children, WHO, 2012), and the incidence of preterm births is approximately 10.6%. The preterm infants birth rate in China ranks second worldwide, accounting for approximately 7.8% of the global rate (Chawanpaiboon et al., 2019). With the development of perinatal medicine and improvement in the treatment of critically ill neonates, preterm infants are increasingly surviving. These preterm infants often develop complications such as bronchopulmonary dysplasia, retinopathy, and intraventricular hemorrhage when discharged from the hospital (Ancel et al., 2015; Bai et al., 2021; Mangiza et al., 2022). Therefore, nursing care for preterm infants after discharge is challenging, and the parents of these infants bear a heavy burden and experience psychological pressure (Linden et al., 2015).

After premature infants are discharged from the hospital, parents need to provide not only the necessary care for their premature infant, such as feeding, bathing, and temperature measurement but should also be able to cope with the provision of some special needs, such as supporting their infants with oxygen inhalation, monitoring their infants to prevent sleep apnea, and performing disease rehabilitation exercises for their infants (Garfield et al., 2014; Connors et al., 2021). Simultaneously, parents also need to acquire the skills for identifying the potential problems of their premature infant after discharge to avoid the occurrence of complications (Treyvaud et al., 2011; Hariati et al., 2021). Mothers may also encounter the stress of caring for a premature baby in addition to coping with recovery from child birth. Studies have shown that mothers of preterm infants experience higher levels of parenting stress after discharge than that experienced by mothers of full-term infants (de Paula et al., 2019; Yao and Zhao, 2020), and this stress persists until the preterm infant is 2 years old (Gray et al., 2017). Parents of preterm infants still experience high levels of anxiety and/or depression with concomitant post-traumatic stress disorder (Schecter et al., 2020) in the first few months after their infants are discharged from the hospital (Ouwendijk-Andréa et al., 2020; Agostini et al., 2022). The stress and anxiety/depression experienced by these parents is caused by difficulties with coping with their premature infant after discharge from the hospital. Nurses need to understand the coping difficulties encountered by parents after discharge and understand their views on nursing needs to adequately prepare parents for the transitioning of their premature infants from the hospital to the family setting (Hua et al., 2020), which will fundamentally reduce the pressure and anxiety/depression of the parents of premature infants. Therefore, it is critical to scientifically and effectively assess the experiences of the parents of premature infants with coping difficulties, rehabilitation management needs, and perceived challenges after discharge.

Post-discharge coping difficulties refer to the physical, psychological, and environmental difficulties faced by individuals, which mainly include stress, postpartum recovery, family management, emotional support, care confidence, post-discharge adaptation, and other difficulties (Weiss et al., 2008; Opper et al., 2019). Adequate preparation for discharge can reduce the coping difficulties of parents after their premature infants are discharged, the readmission rate, and the number of emergency department visits (Weiss et al., 2017, 2020). The English version of the Ped-PDCDS was developed by Professor Weiss in 2008 to measure the coping difficulties faced by parents 3 weeks after their hospitalized preterm children have been discharged (Weiss et al., 2008) and coping difficulties of the parents of children discharged from the neonatal intensive care unit (NICU). Ped-PDCDS has been used in Germany, Norway, Turkey, and other countries (Marquette University, 2022) but has not been used in China. In this study, the English version of the Ped-PDCDS was translated into Chinese, and its reliability and validity in preterm infants were further verified to provide a reliable evaluation tool for measuring the coping difficulties of Chinese parents of preterm infants after discharge.

## 2. Materials and methods

### 2.1. Study design

Our research team had established a multi-center prospective observational cohort of preterm discharge readiness since May 2022. This cohort recruited five tertiary hospitals in Shandong Province, China as partners. All five NICUs admitted critically ill neonates who were on mechanical ventilation. NICU-1 had 40 beds, NICU-2 had 60 beds, NICU-3 had 78 beds, NICU-4 had 40 beds, and NICU-5 had 30 beds. On average, more than 800 infants were admitted to these hospitals each year. All data in this study came from this cohort. Data collection was performed between May and September 2022.

### 2.2. Participants and data collection

The inclusion criteria were as follows: (a) parents of premature infants with a gestational age of less than 37 weeks at admission. (b) parents older than 18 years; (c) who provided informed consent; and (d) who were primary caregivers of preterm infants. The exclusion criteria were as follows: (a) parents with cognitive impairment; (b) parents of premature infants who died during hospitalization; and (c) parents of premature infants who automatically discharged/transferred during hospitalization.

A nurse was selected from each center to participate in the survey after obtaining consent from the head nurse of each center. Before the survey, the researchers trained the nurses, provided information on the time and method of the survey, and used unified guidelines for addressing problems that might be encountered during the survey. Three weeks after the preterm infants were discharged from the hospital, the nurses called the primary caregivers of infants, and the nurses explained to them the purpose of the study. We surveyed all parents of preterm infants after 3 weeks of discharge. To improve the response rate of parents, the nurses asked the parents about the problems they encounter when providing care for their infants and helped them solve these problems as much as possible. Subsequently, an electronic version (Sojump) of the questionnaire was sent to the primary caregivers.

### 2.3. Ethical considerations

Ethical approval was obtained from the ethics committee of Shandong Provincial Hospital Affiliated to Shandong First Medical University, China (SWYX: NO.2022–366). In accordance with the ethical principles of the Declaration of Helsinki, the privacy and personal information of parents and premature infants was kept confidential, and all participating parents signed written informed consent forms.

### 2.4. Post-discharge coping difficulty scale-parent form

The scale was developed by Professor Weiss in 2008 (Weiss et al., 2008) based on the Coping Difficulties Scale for adult patients after discharge. It was specially designed to measure the coping difficulties of the parents of hospitalized children (including those in NICU) after discharge. The scale has 11 items. The follow-up survey, which mainly measures the coping difficulties of parents after discharge, including coping with stress, rehabilitation, and disease management difficulties; need for help and emotional support; and confidence in and adaptation to child care and medical management, is usually conducted between 2 and 3 weeks after the infants are discharged. Items 1–5 have subitems, which are open-ended questions and are not scored. Item 6b is a scoring question but is not counted as part of the total score. The scale is scored from 0 to 10, with 0 representing “none at all” and 10 representing “extremely/a great deal/completely.” Items 8, 9, 10a, and 10b are reverse scored. To obtain a total score, the items are summed, and the sum is divided by the number of items in the scale. The final score is the mean score of the items and ranges from 0 to 10; the higher the score, the more difficulties the parents faced after the child was discharged.

### 2.5. Translation process

After the researcher contacted Professor Weiss for authorization and consent, the English version of the Ped-PDCDS was translated into Chinese using the translation method described in the study by Sousa and Rojjanasrirat (2011). Step 1: Two translators with good bilingual background were invited to independently translate the English version to the Chinese version. One translator, whose native language is Chinese and who studied in the United States for 5 years, has a PhD in nursing, and the other, who studied and worked in Singapore for 10 years, is an English lecturer. Step 2: The two translated versions were synthesized by the research team, including two nursing tutors, one pediatric medical doctor with 15 years of experience, and one pediatric nursing manager with a master’s degree in nursing, to develop Chinese version 1. Step 3: Two translators, who had never been exposed to the original scale, were invited to independently translate Chinese version 1 into English. Both translators were native Chinese speakers. One translator is a doctor of nursing and is engaged in nursing education in the United States, and the other is a doctor of nursing who studied in the United States for 2 years. Step 4: The research team compared the two versions to form the back-translated version 1. Step 5: The back-translated version 1 was sent to the original author of the scale. Based on the professor’s opinion of the back-translated version, the objective items were discussed and modified, and Chinese version 2 was finally developed.

### 2.6. Cultural adaptation

Six experts, including two neonatal clinical experts, three neonatal nursing managers, and one neonatal specialist nurse, were invited to form an expert group to evaluate the clarity of expression, accuracy of content and importance of Chinese version 2. Their average age was 37.6 ± 7.5 (range, 33–52) years and years of experience was 15.9 ± 9.0 (range, 7–33) years. Three of the experts were master’s supervisors, and three were doctoral supervisors. We calculated the authority coefficient (Cr) (Dai et al., 2019), which is used to measure the reliability and representativeness of expert consultations of the experts. The judgment criteria (Ca) score of the experts was 0.90, and the familiarity degree (Cs) of the experts with the questionnaire items was 0.84. Therefore, Cr = (Cs + Ca)/2 = 0.87, and the response rate of the experts was 100%. The research team revised Chinese version 2 according to the opinions of the experts and finally obtained Chinese version 3.

### 2.7. Pilot testing

The convenience sampling method was used to select 30 parents who met the inclusion and exclusion criteria in one of the five hospitals. Before the survey, the significance of the survey was explained to the parents. Simultaneously, Ped-PDCDS Chinese Version 3 was sent to the parents through a questionnaire star 3 weeks after their infants were discharged from the hospital, and the parents were also asked how they understood the scale and their experience with filling the form through a phone call. The researchers recorded the filling time, problems with the form, and suggestions. The final Chinese version of the Ped-PDCDS was developed.

### 2.8. Statistical analysis

IBM SPSS 24.0 software was used for statistical analysis, and Amos 26.0 software was used for confirmatory factor analysis.

The correlation coefficient and decision value were used to determine the items that should be excluded from the scale. Correlation coefficient method: the correlation coefficient between the score of each item and the total score of the scale was calculated. If the correlation coefficient was <0.4 or the *p* value was >0.05, the item was excluded from the scale (Terwee et al., 2007). Decision value method: the total score of the scale was ranked from high to low, and the 27% limit of the high and low scores was selected. The first 27% was the high group, and the last 27% was the low group. Two independent samples t-test was used for comparisons between the two groups, and the decision value of each item was calculated. If the p value of an item was >0.05 or the decision value of an item was <3.00, the item was excluded from the scale (Hu and Wang, 2022).

Seven experts were invited to evaluate the content validity of the scale. The Likert 4-level scoring method (1 = no correlation, 2 = weak correlation, 3 = strong correlation, and 4 = very correlation) was used to evaluate the correlation between each item and the total scale. The item-level content validity index (I-CVI) and scale-level content validity index (S-CVI) was calculated. The I-CVI ≥0.78 and S-CVI ≥0.80 indicated acceptable content validity indexes (Polit and Beck, 2006).

The construct validity of the Chinese version of the Ped-PDCDS was measured using exploratory factor analysis (EFA) and confirmatory factor analysis (CFA). Principal component analysis and the maximum variance orthogonal rotation method were used for the EFA. Kaiser-Meyer-Olkin (KMO) and Bartlett’s sphericity tests were performed to investigate whether the items of the scale are suitable for EFA. If the KMO value was >0.8 and the Bartlett’s sphericity test was significant (*p* < 0.05), it indicated that the items were suitable for factor analysis (Li and Liu, 2018). CFA uses the maximum likelihood method to test the fit degree of the model. The ratio of chi-square to the degrees of freedom (x^2^/df), root mean square error of approximation (RMSEA), goodness of fit index (GFI), comparative fit index (CFI), normed fit index (NFI), incremental fit index (IFI), non-standard fit index (TLI) and increasing fit index (IFI) were used to evaluate the model. Factor loading, combination reliability, and average variance extracted (AVE) were used to determine the convergent and divergent validities. If the factor loading is >0.50, combination reliability is >0.70, and AVE is >0.50, it indicates that the variables have good convergent validity. If there is a significant correlation between the variables and the correlation is lower than the square root of the AVE value, it indicates that the variables have good discriminant validity (Souza et al., 2017).

Cronbach’s alpha coefficient, split-half reliability, and test–retest reliability were calculated to evaluate the reliability of the scale. Pearson’s correlation coefficient was used to correlate the test and test–retest scores to evaluate the test–retest reliability of the scale. The Cronbach’s alpha coefficient of the scale was >0.70, the split-half reliability was >0.8 (Hu and Wang, 2022), and Pearson’s correlation coefficient was >0.70 (Terwee et al., 2007), indicating that the reliability of the scale is acceptable.

## 3. Results

### 3.1. Participant characteristics

The questionnaire was sent to each of the 380 parents of premature infants, 356 of which were filled and returned, with an effective response rate of 93.8%. Among them, 299 (83.9%) were mothers, and the remaining were fathers. The average age was 31.9 ± 5.3 (range, 21–49) years, 351 (98.7%) were married, and 166 (46.6%) lived in rural areas. The gestational age of the preterm infants was 33.2 ± 2.8 (range, 24.7–36.9) weeks, birth weight was 580–4,500 g (mean, 1995.7 ± 654.3 g), 195 (54.7%) were boys, 311 (87.4%) were singleton deliveries, and the length of hospital stay was 25.9 ± 22.3 (range 5–126) days.

### 3.2. Content validity

Seven experts (five master’s degree and two doctoral students), including two clinical nurses, four neonatal nursing managers, and one nursing educator, were invited to evaluate the content validity. All of them were female and aged 39.6 ± 6.5 (range, 34–52) years. The average years of work experience was 14.9 ± 8.0 (range, 10–33) years. The I-CVI ranged from 0.86 to 1.00, and the S-CVI was 0.98.

### 3.3. Item analysis

The results of the correlation analysis showed that the correlation coefficient between the score of each item and the total score of the scale was 0.43–0.80. The decision value analysis results between the high and low groups was 5.91–21.11, and the difference was statistically significant (*p* < 0.001). The item analysis results are shown in Table 1.

**Table 1:** Pearson’s correlation coefficient between each item and the total score of the questionnaire.

Items	Mean value	Standard deviation	*R*
A1 How stressful has your life been?	3.76	3.19	0.70*
A2 How much difficulty have you had with your child’s recovery?	2.58	2.77	0.69*
A3 How much difficulty have you had with caring for your child?	3.05	2.82	0.80*
A4 How much difficulty have you had with managing your child’s medical condition?	2.34	2.74	0.71*
A5 How difficult has the time been for your family members or other close persons?	2.62	2.86	0.71*
B6a How much help have you needed with caring for your child?	4.58	3.52	0.61*
B6b How much help had you expected to need?	4.81	3.51	0.60*
B7 How much emotional support have you needed?	6.14	3.72	0.50*
C8 How confident have you felt in your ability to care for your child’s needs?	2.97	2.70	0.45*
C9 Have you been able to take care of your child’s medical needs such as medications or treatments?	2.76	2.92	0.43*
C10a How well have you adjusted to your child being at home since your child’s hospitalization?	2.05	2.28	0.59*
C10b How well has your child adjusted to being at home after discharge from the hospital?	1.87	2.20	0.52*

*p < 0.01.

### 3.4. EFA

A total of 178 cases were randomly selected from the sample for EFA. The KMO value was 0.83, and the Bartlett’s sphericity test *χ*^2^ value was 1344.14 (df = 66, *p* < 0.001), indicating that the scale could be used for EFA. The principal component analysis and variance maximum orthogonal rotation methods were used to extract common factors with an eigenvalue >1. The following three dimensions were extracted from the scale: dimension 1, named difficulty perception; dimension 2, support requirement; and dimension 3, coping efficacy. The cumulative variance contribution rate was 73.12%. The factor loading of the items was 0.73–0.90. The results of the EFA are shown in Table 2.

**Table 2:** Factor loadings of the Chinese version Ped-PDCDS.

Items	F1	F2	F3	Common factor variance
A1 How stressful has your life been?	**0.79**	0.14	0.26	0.72
A2 How much difficulty have you had with your child’s recovery?	**0.88**	0.09	0.09	0.79
A3 How much difficulty have you had with caring for your child?	**0.85**	0.16	0.27	0.82
A4 How much difficulty have you had with managing your child’s medical condition?	**0.81**	0.10	0.15	0.69
A5 How difficult has the time been for your family members or other close persons?	**0.73**	0.18	0.20	0.61
B6a How much help have you needed with caring for your child?	0.25	0.04	**0.90**	0.87
B6b How much help had you expected to need?	0.25	0.08	**0.89**	0.86
B7 How much emotional support have you needed?	0.19	0.00	**0.83**	0.72
C8 How confident have you felt in your ability to care for your child’s needs?	0.03	**0.76**	0.09	0.59
C9 Have you been able to take care of your child’s edical needs such as medications or treatments?	0.16	**0.75**	−0.03	0.59
C10a How well have you adjusted to your child being at home since your child’s hospitalization?	0.24	**0.87**	0.08	0.81
C10b How well has your child adjusted to being at home after discharge from the hospital?	0.12	**0.88**	0.01	0.79
KMO value	0.83			
Barthes spherical value	1344.14			
df	66			
*p*- value	0			

F1, difficulty perception; F2, support requirement; F3,coping efficacy. The bold values mean factor loadings of the item in its respective dimension.

### 3.5. CFA

**Figure 1:**
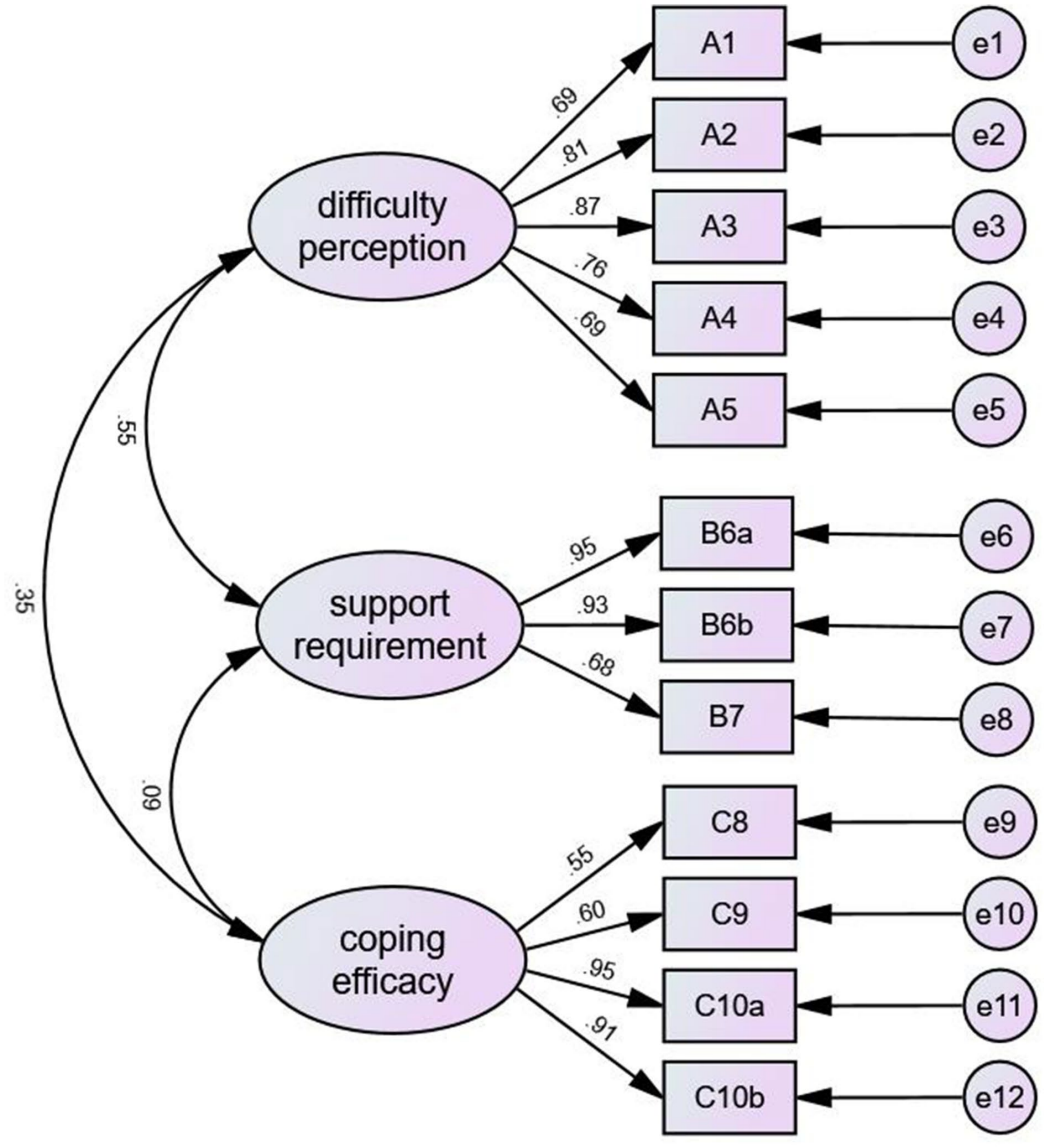
The results of CFA.

A total of 178 cases were randomly selected from the sample for CFA We performed a CFA of the three factors based on the results from the EFA and found that the model had a good fit (x^2^/df = 2.35, RMSEA = 0.06, GFI = 0.95, CFI = 0.97, NFI = 0.95, IFI = 0.97, and TLI = 0.97). The results of CFA are shown in Figure 1, and the fitting index of the model is shown in Table 3.

**Table 3:** Evaluation of the goodness-of-fit of the model.

Item	*x*^2^/df	RMSEA	GFI	CFI	NFI	IFI	TLI
Model value	2.35	0.06	0.95	0.97	0.95	0.97	0.97
Suggested value	<3	<0.08	>0.9	>0.9	>0.9	>0.9	>0.9

### 3.6. Convergent and discriminant validities

We calculated the composite reliability (CR) and AVE values of the questionnaire. The CR values of the three dimensions ranged from 0.59 to 0.74, and the AVE values ranged from 0.85 to 0.89 (Table 4). Table 5 shows the discriminant validity of the Chinese version. The square root of the AVE was greater than the correlation coefficient between each dimension.

**Table 4:** Results of the convergent validity analysis.

Relationship between variables			Estimated coefficient	Standard error	Critical value	*p*	Load factor	AVE	Combination reliability
A1	<−--	Difficulty perception	1			*	0.70	0.88	0.59
A2	<−--	Difficulty perception	0.99	0.07	13.76	*	0.81		
A3	<−--	Difficulty perception	1.11	0.08	14.61	*	0.87		
A4	<−--	Difficulty perception	0.93	0.07	13.02	*	0.76		
A5	<−--	Difficulty perception	0.88	0.07	11.93	*	0.69		
B6a	<−--	Support requirement	1.00			*	0.95	0.89	0.74
B6b	<−--	Support requirement	0.97	0.04	27.07	*	0.93		
B7	<−--	Support requirement	0.74	0.05	15.63	*	0.68		
C8	<−--	Coping efficacy	1.00			*	0.55	0.85	0.60
C9	<−--	Coping efficacy	1.18	0.13	9.02	*	0.60		
C10a	<−--	Coping efficacy	1.45	0.13	11.39	*	0.95		
C10b	<−--	Coping efficacy	1.33	0.12	11.34	*	0.91		

^*^p < 0.01.

**Table 5:** Results of the discriminant validity analysis using the Pearson’s coefficient.

	Difficulty perception	Support requirements	Coping efficacy
Difficulty perception	0.88		
Support requirements	0.55*	0.89	
Coping efficacy	0.35*	0.09	0.85
AVE square root	0.94	0.94	0.92

^*^p < 0.01.

### 3.7. Reliability

The Cronbach’s alpha coefficient of the dimensions 1, 2, and 3 of the Chinese version of the Ped-PDCDS scale was 0.88, 0.89, and 0.86, respectively. Thirty participants were randomly selected from the sample and were further followed up by a telephone call 5 weeks after discharge (the interval between the two assessments was 14 days) to evaluate the test–retest reliability of the Chinese version of the Ped-PDCDS. The overall reliability of the scale was 0.85, the split-half reliability was 0.92, and the test–retest reliability was 0.84. The reliability results of the scale are shown in Table 6.

**Table 6:** Reliability of the Chinese version Ped-PDCDS.

Dimensionality	Cronbach’s α coefficient	Test–retest reliability	Split-half reliability
Difficulty perception	0.88	0.80*	0.81
Support requirements	0.89	0.81*	0.82
Coping efficacy	0.86	0.80*	0.89
The scale	0.85	0.84*	0.92

^*^p < 0.01.

## 4. Discussion

The patient’s parents, as the close relative, bore more burden of care (Li et al., 2021), and often had difficulties in coping with the care burden. After premature infants are discharged from the hospital, parents, as the primary caregivers, bore more burden of care, such as feeding, taking oral medication, and temperature measurement et al. Different from taking care of full-term infants, premature infants often developed complications of respiratory and digestive systems, and even intracranial hemorrhage when discharged from the hospital (Ancel et al., 2015). Therefore, premature infants needed oxygen inhalation, rehabilitation exercises or stoma care after returning home. The medical related care was a challenge for parents, and so that parents would find it difficult to cope.

The measurement of coping difficulties after discharge provides objective data that can guide nurses to adequately prepare patients for discharge (Miller et al., 2008; Li et al., 2022). After translation and cultural adaptation, and reliability and validity tests, the Chinese version of the Ped-PDCDS scale finally included 11 items, and the questionnaire completion time was 3–5 min. This questionnaire showed semantic and cultural equivalence, had satisfactory reliability and validity, and could be popularized in clinical practice.

In the item analysis, all the relationship coefficients between each item and the total scale were > 0.4, indicating that each item had high homogeneity with the total scale (Terwee et al., 2007). The decision value of each item was >3.0, and the difference was statistically significant (*p* < 0.001), indicating that each item of the scale had a good discriminative ability (Hu and Wang, 2022). Therefore, all the 11 items were retained. Reliability reflects the consistency of the scale. The higher the reliability, the higher the accuracy of the scale (Hu and Wang, 2022). The results of this study showed that the Cronbach’s alpha coefficient between each dimension and the overall scale was >0.8, which was higher than that of the original scale (Weiss et al., 2008), indicating that the scale had good internal consistency. The correlation between each dimension and the test–retest reliability of the scale ranged from 0.80 to 0.84, indicating that the scale had good temporal stability.

Validity measures the extent to which the scale truly reflects the concept it is intended to measure (Mokkink et al., 2010). In this study, EFA and CFA were used to verify the validity of the scale. The EFA results showed that the Chinese version of the Ped-PDCDS had three dimensions, the factor loading of the items ranged from 0.68 to 0.90, and the cumulative variance contribution rate was 73.12%; all these results were within acceptable ranges. The factor loading was >0.4, and the cumulative variance contribution rate was >50% (Li and Liu, 2018), indicating that the construct validity of the scale was acceptable. The CFA results showed that x^2^/df was <3.0, RMSEA was <0.08, and GFI, CFI, NFI, IFI, TLI, and IFI were > 0.9. The factor loadings between the items and each dimension ranged from 0.55 to 0.95, indicating that the three dimensions of the Chinese version of the Ped-PDCDS were reasonable (Li and Liu, 2018). This is different from the original scale structure, which may be caused by the inconsistency with the research participants and differences between the Chinese and Western cultures. In addition, the convergent and discriminant validities of the scale were analyzed. The AVE values of each dimension ranged from 0.85 to 0.89, CR ranged from 0.59 to 0.74, and the square root of AVE was greater than the correlation coefficient between each dimension. The results indicated that the convergent and discriminant validities of the scale were satisfactory.

Considering the good model fitting and experts’ opinions, three common dimensions were finally extracted in this study. According to the stress and coping model of Folkman and Lazarus (1985) and Folkman et al. (1986), when the coping resources of people cannot meet their internal and external needs, stress is generated. Subsequently, these individuals evaluate the stress, such as the cognition of stress, response to stress and self-efficacy. The three dimensions derived from the Chinese version of the Ped-PDCDS were consistent with this theoretical coping model. Parents of preterm infants encounter great stress and negative emotions after their children are discharged from the hospital (Linden et al., 2015; Lehtonen et al., 2022). The first dimension of the scale (items 1–5), named difficulty perception, evaluates pressure perception, which mainly refers to the pressure parents feel and difficulties they encounter, including daily life pressure and daily nursing, disease management, and rehabilitation exercise difficulties, during the care of their premature infants after discharge. When parents encounter pressure and difficulties, they adopt certain strategies, such as need support and help to cope (Malliarou et al., 2021). The second dimension of the scale (items 6a, 6b, and 7), named support requirement, measures parents’ need for help and emotional support, which are some responses to stress. The third dimension (Items 8–10b), named self-efficacy, refers to the ability and confidence of an individual to accomplish a certain goal (Lei et al., 2020). This dimension measures parental confidence in child care, the extent to which children’s medical needs are met, and the resilience of both children and parents at home.

In line with the original scale, the Chinese version of the Ped-PDCDS scale includes additional open-ended items alongside the 1–5 scale items, which do not impact the overall scoring of the scale. These supplementary items are designed to capture specific life stressors experienced by parents of premature infants, as well as the unique challenges they face in their child’s recovery, caregiving, and medical issues. These open-ended questions serve as supplementary measures to provide researchers with a more comprehensive and detailed understanding of the difficulties encountered by parents after their premature infant’s discharge. The analysis approach for these open-ended items depends on the research objectives and design. If the goal of utilizing the scale is to gain deeper insights into participants’ perspectives and experiences, it is recommended that researchers perform qualitative analysis of the responses. If there is a need to quantify specific aspects of the open-ended responses, researchers can assign responses to different categories and calculate the frequency or proportion within each category. Regardless of the chosen analysis method, it is advisable to involve at least two independent researchers in the analysis process and conduct cross-validation when necessary to ensure the reliability and consistency of the findings.

Data of the study were collected from the multi-center prospective observational cohort of preterm discharge readiness. The sample was sufficient. The two-way translation was strictly adopted in the translation process, and the model fit of the Chinese Ped-PDCDS was satisfactory. The three dimensions extracted from the scale were consistent with the Chinese cultural background. Our study also has some limitations. In this study, primary caregivers of premature infants after discharge were selected for investigation. In China’s traditional family setting, mothers are the primary caregivers, so a large proportion of the participants in this study were mothers. The population assessed when using the English version of the Ped-PDCDS is mainly parents of hospitalized children within a wide age range whose diseases are complex. Although this study is a multi-center study, the selected sample were parents of hospitalized preterm infants, which limits the application of the Chinese version of the Ped-PDCDS in other hospitalized children. In the future, we will expand the sample population range of the survey to further verify the applicability of the Chinese version in other hospitalized children.

## 5. Conclusion

Psychological measurements of the Chinese version of the Ped-PDCDS among parents of preterm infants were performed in this study. This study tested the reliability and validity of the scale, and the results were satisfactory. This scale is simple and easy to understand, and less time is required to complete a questionnaire; therefore, it can be widely used to measure the coping difficulties faced by parents of premature infants after the infants are discharged.

## Data availability statement

The original contributions presented in the study are included in the article/supplementary material, further inquiries can be directed to the corresponding author.

## Ethics statement

Ethical approval was obtained from the ethics committee of Shandong Provincial Hospital Affiliated to Shandong First Medical University, China (SWYX: NO.2022-366). In accordance with the ethical principles of the Declaration of Helsinki, the privacy and personal information of parents and premature infants was kept confidential, and all participating parents signed written informed consent forms.

## Author contributions

JL, ML, and ZS contributed to the study design, implementation, analysis, and manuscript writing. JJG and QS contributed to the study design and data analysis. JLG, LX, XL, and YS contributed to the collection of the data. All authors contributed to the article and approved the submitted version.

## Funding

This work was supported by Shandong Provincial Traditional Chinese Medicine Science and Technology Project (grant number M-2023164).

## Conflict of interest

The authors declare that the research was conducted in the absence of any commercial or financial relationships that could be construed as a potential conflict of interest.

## Publisher’s note

All claims expressed in this article are solely those of the authors and do not necessarily represent those of their affiliated organizations, or those of the publisher, the editors and the reviewers. Any product that may be evaluated in this article, or claim that may be made by its manufacturer, is not guaranteed or endorsed by the publisher.

## References

[ref1] Agostini F. Neri E. Genova F. Trombini E. Provera A. Biasini A.. (2022). Depressive symptoms in fathers during the first postpartum year: the influence of severity of preterm birth, parenting stress and partners’ depression. Int. J. Env. Res. Pub. He. 19:9478. doi: 10.3390/ijerph19159478, PMID: 35954832 PMC9368501

[ref2] Ancel P. Goffinet F. Kuhn P. Langer B. Matis J. Hernandorena X.. (2015). Survival and morbidity of preterm children born at 22 through 34 weeks' gestation in France in 2011. JAMA Pediatr. 169, 230–238. doi: 10.1001/jamapediatrics.2014.3351, PMID: 25621457

[ref3] Bai R. Jiang S. Guo J. Jiang S. Lee S. K. Li Z.. (2021). Variation of neonatal outcomes and care practices for preterm infants <34 weeks' gestation in different regions of China: a cohort study. Front. Pediatr. 9:760646. doi: 10.3389/fped.2021.760646, PMID: 34869117 PMC8636048

[ref4] Chawanpaiboon S. Vogel J. P. Moller A. Lumbiganon P. Petzold M. Hogan D.. (2019). Global, regional, and national estimates of levels of preterm birth in 2014: a systematic review and modelling analysis. Lancet Glob. Health 7, e37–e46. doi: 10.1016/S2214-109X(18)30451-0, PMID: 30389451 PMC6293055

[ref5] Connors J. Havranek T. Campbell D. (2021). Discharge of medically complex infants and developmental follow-up. Pediatr. Rev. 42, 316–328. doi: 10.1542/pir.2020-000638, PMID: 34074718

[ref6] Dai F. Wei K. Chen Y. Ju M. (2019). Construction of an index system for qualitative evaluation of undergraduate nursing students innovative ability: a Delphi study. J. Clin. Nurs. 28, 4379–4388. doi: 10.1111/jocn.15020, PMID: 31411352

[ref7] de Paula E. J. de Rezende M. G. Menezes P. R. Del-Ben C. M. (2019). Preterm birth as a risk factor for postpartum depression: a systematic review and meta-analysis. J. Affect. Disord. 259, 392–403. doi: 10.1016/j.jad.2019.08.069, PMID: 31470184

[ref8] Folkman S. Lazarus R. S. (1985). If it changes it must be a process: study of emotion and coping during three stages of a college examination. J. Pers. Soc. Psychol. 48, 150–170. doi: 10.1037//0022-3514.48.1.150, PMID: 2980281

[ref9] Folkman S. Lazarus R. S. Dunkel-Schetter C. DeLongis A. Gruen R. J. (1986). Dynamics of a stressful encounter: cognitive appraisal, coping, and encounter outcomes. J. Pers. Soc. Psychol. 50, 992–1003. doi: 10.1037//0022-3514.50.5.992, PMID: 3712234

[ref10] Garfield C. F. Lee Y. Kim H. N. (2014). Paternal and maternal concerns for their very low-birth-weight infants transitioning from the NICU to home. J. Perinat. Neonat. Nur. 28, 305–312. doi: 10.1097/JPN.0000000000000021, PMID: 24927295

[ref11] Gray P. H. Edwards D. M. Gibbons K. (2017). Parenting stress trajectories in mothers of very preterm infants to 2 years. Arch Dis Child Fetal Neonatal Ed 103, F43–F48. doi: 10.1136/archdischild-2016-312141 28659361

[ref12] Hariati S. Sutomo R. McKenna L. Reisenhofer S. Lusmilasari L. Febriani A. D. B. (2021). Indonesian mothers' beliefs on caring practices at home for preterm babies after hospital discharge: a qualitative study. J. Spec. Pediatr. Nurs. 26:e12330. doi: 10.1111/jspn.12330, PMID: 33773015

[ref13] Hu Y. Wang Z. (2022). Chinese nursing research 6th. Beijing, China: People'Medical Publishing House.

[ref14] Hua W. Yuwen W. Simoni J. M. Yan J. Jiang L. (2020). Parental readiness for hospital discharge as a mediator between quality of discharge teaching and parental self-efficacy in parents of preterm infants. J. Clin. Nurs. 29, 3754–3763. doi: 10.1111/jocn.15405, PMID: 32644290

[ref15] Lehtonen L. Lilliesköld S. De Coen K. Toome L. Gimeno A. Caballero S.. (2022). Parent–infant closeness after preterm birth and depressive symptoms: a longitudinal study. Front. Psychol. 13:906531. doi: 10.3389/fpsyg.2022.906531, PMID: 36237668 PMC9551610

[ref16] Lei H. Yuan Y. Wang Z. Chen J. Li Z. (2020). Measurement invariance and intergenerational parallelism of general self-efficacy in adolescent and parent dyads. Front. Psychol. 11:1251. doi: 10.3389/fpsyg.2020.01251 32581980 PMC7287210

[ref17] Li L. Z. Bian J. Y. Wang S. (2021). Moving beyond family: unequal burden across mental health patients’ social networks. Qual. Life Res. 30, 1873–1879. doi: 10.1007/s11136-021-02782-9, PMID: 33566303

[ref18] Li Z. Liu Y. (2018). Nursing research methods 2th. Beijing: People's Medical Publishing House.

[ref19] Li W. Zhang Y. Wei L. (2022). Postdischarge coping difficulties and influencing factors of pregnant women with gestational diabetes mellitus: a cross-sectional study. Nurs. Open 9, 1625–1634. doi: 10.1002/nop2.1186, PMID: 35240006 PMC8994934

[ref20] Linden M. A. Cepeda I. L. Synnes A. Grunau R. E. (2015). Stress in parents of children born very preterm is predicted by child externalising behaviour and parent coping at age 7 years. Arch. Dis. Child. 100, 554–558. doi: 10.1136/archdischild-2014-307390, PMID: 25762532

[ref21] Malliarou M. Karadonta A. Mitroulas S. Paralikas T. Kotrotsiou S. Athanasios N.. (2021). Preterm parents' stress and coping strategies in a neonatal intensive care unit in a university hospital of Central Greece. Materia Socio Medica. 33, 244–249. doi: 10.5455/msm.2021.33.244-249, PMID: 35210945 PMC8812370

[ref22] Mangiza M. Ehret D. E. Y. Edwards E. M. Rhoda N. Tooke L. (2022). Morbidity and mortality in small for gestational age very preterm infants in a middle-income country. Front. Pediatr. 10:915796. doi: 10.3389/fped.2022.915796, PMID: 36016879 PMC9396138

[ref23] March of Dimes, PMNCH, Save The Children, WHO (2012). Born too soon: the global action report on preterm birth. Geneva: World Health Organization.

[ref24] Marquette University (2022). Hospital discharge scales -- post-discharge coping difficulty scale (pdcds). Available at: https://www.marquette.edu/nursing/hospital-discharge-scales-post-discharge-coping.php (Accessed October 15, 2022).

[ref25] Miller J. F. Piacentine L. B. Weiss M. (2008). Coping difficulties after hospitalization. Clin. Nurs. Res. 17, 278–296. doi: 10.1177/1054773808325226 18927261

[ref26] Mokkink L. B. Terwee C. B. Patrick D. L. Alonso J. Stratford P. W. Knol D. L.. (2010). The COSMIN study reached international consensus on taxonomy, terminology, and definitions of measurement properties for health-related patient-reported outcomes. J. Clin. Epidemiol. 63, 737–745. doi: 10.1016/j.jclinepi.2010.02.006, PMID: 20494804

[ref27] Opper K. Beiler J. Yakusheva O. Weiss M. (2019). Effects of implementing a health team communication redesign on hospital readmissions within 30 days. Worldv. Evid. Based Nu. 16, 121–130. doi: 10.1111/wvn.12350, PMID: 30919571

[ref28] Ouwendijk-Andréa M. Bröring-Starre T. Molderink A. C. Laarman C. A. R. C. Oostrom K. J. van Dijk-Lokkart E. M. (2020). Parental emotional distress after discharge from the neonatal intensive care unit: a pilot study. Early Hum. Dev. 140:104892. doi: 10.1016/j.earlhumdev.2019.104892 31715521

[ref29] Polit D. F. Beck C. T. (2006). The content validity index: are you sure you know what's being reported? Critique and recommendations. Res. Nurs. Health. 29, 489–497. doi: 10.1002/nur.20147, PMID: 16977646

[ref30] Schecter R. Pham T. Hua A. Spinazzola R. Sonnenklar J. Li D.. (2020). Prevalence and longevity of PTSD symptoms among parents of NICU infants analyzed across gestational age categories. Clin. Pediatr. 59, 163–169. doi: 10.1177/0009922819892046, PMID: 31833404

[ref31] Sousa V. D. Rojjanasrirat W. (2011). Translation, adaptation and validation of instruments or scales for use in cross-cultural health care research: a clear and user-friendly guideline. J. Eval. Clin. Pract. 17, 268–274. doi: 10.1111/j.1365-2753.2010.01434.x 20874835

[ref32] Souza A. C. D. Alexandre N. M. C. Guirardello E. D. B. Souza A. C. D. Alexandre N. M. C. Guirardello E. D. B. (2017). Propriedades psicométricas na avaliação de instrumentos: Avaliação da confiabilidade e da validade. Epidemiol Serv Saúde. 26, 649–659. doi: 10.5123/S1679-49742017000300022, PMID: 28977189

[ref33] Terwee C. B. Bot S. D. M. de Boer M. R. van der Windt D. A. W. M. Knol D. L. Dekker J.. (2007). Quality criteria were proposed for measurement properties of health status questionnaires. J. Clin. Epidemiol. 60, 34–42. doi: 10.1016/j.jclinepi.2006.03.012 17161752

[ref34] Treyvaud K. Doyle L. W. Lee K. J. Roberts G. Cheong J. L. Y. Inder T. E.. (2011). Family functioning, burden and parenting stress 2 years after very preterm birth. Early Hum. Dev. 87, 427–431. doi: 10.1016/j.earlhumdev.2011.03.008, PMID: 21497029

[ref35] Weiss M. Johnson N. L. Malin S. Jerofke T. Lang C. Sherburne E. (2008). Readiness for discharge in parents of hospitalized children. J. Pediatr. Nurs. 23, 282–295. doi: 10.1016/j.pedn.2007.10.005 18638672

[ref36] Weiss M. E. Lerret S. M. Sawin K. J. Schiffman R. F. (2020). Parent readiness for hospital discharge scale: psychometrics and association with postdischarge outcomes. J. Pediatr. Health Car. 34, 30–37. doi: 10.1016/j.pedhc.2019.06.011, PMID: 31575440

[ref37] Weiss M. E. Sawin K. J. Gralton K. Johnson N. Klingbeil C. Lerret S.. (2017). Discharge teaching, readiness for discharge, and post-discharge outcomes in parents of hospitalized children. J. Pediatr. Nurs. 34, 58–64. doi: 10.1016/j.pedn.2016.12.021 28087088

[ref38] Yao L. Zhao M. (2020). Research progress on parenting stress of very low birth weight premature infants after discharge from hospital. Chin J Mod Nurs. 26, 4707–4711. doi: 10.3760/cma.j.cn115682-20200116-00244

